# Unleashing Potential of a Medical Student in a Medical Journal: Dual Full-Time Responsibilities

**DOI:** 10.31729/jnma.6544

**Published:** 2021-05-31

**Authors:** Asmita Neupane

**Affiliations:** 1 Kathmandu Medical College and Teaching Hospital, Sinamangal, Kathmandu, Nepal

**Keywords:** *assistant editor*, *journal*, *medical student*

## Abstract

"Only those who will risk going too far can possibly find out how far one can go"-TS Elliot. Joining the editorial support as a medical student, at the Journal of Nepal Medical Association, full-time responsibilities, were challenging to perform. Applying and getting into the Journal of Nepal Medical Association team was only the start of a very vast learning experience. Learning leadership qualities, time management skills, communication skills and technical skills, helped me to excel in my duty as assistant editor and a medical student. Involving in the training and working with authors and the editorial team, I have learned about the actual research and its scenario in Nepal.

## INTRODUCTION

Starting medical school is challenging and to thrive is more challenging. It is not just the study: it is about the prospect of meeting new people, adapting to a different lifestyle and place, and managing independence and time. Particularly in the early stages of medical school, you might feel as if you're oscillating between two extremes: when all you do is work and rush around, and when you'll go out, socialise, and the last thing you'll want to do is open a textbook or go to a lecture or do revisions.^1^

Being able to manage your time effectively is one of the key skills you'll learn while working as a medical student and assistant editor. Since, most student medical editors work part-time, sharing their editorial work with other research and their education. Try to stick to a schedule, and plan and utilize your time carefully around work, extracurricular activities, and social commitments.^1^ Both of the jobs are equally demanding and require a different set of skills to excel at both of them.

## GETTING INTO THE TEAM

I joined research training for a month from July 2018 to August 2018 as a participant at the Journal of Nepal Medical Association (JNMA). After finishing my classes at my medical college, I used to reach the office of JNMA for the training. On average, our training session used to last for 3 hours. During our training, the opportunity to join the JNMA editorial team was given, seizing the opportunity I joined the team as a JNMA trainee. After filling up the forms and interviews, entering the washout period, and completing of the personal task, I finally entered the JNMA team in the post of editorial support. Even after giving multiple interviews at school magazine, college magazine and journals, I never got the chance to work as an editor. Dream of being an editor, this opportunity at JNMA brought me closer to my dream. The hardest part of the whole selection process was the completion of the personal task that pushed my limits, threw me out of the comfort zone and made me realize, with determination, anything is achievable.

## WORKING WITH THE TEAM

Editors are often visualized sitting at a desk struggling over one manuscript after another. They do that, but they do far more.^1^ So as a part of the editorial team, which started with the editing of basic format articles submitted for publication in JNMA. Later over the time, it included working on OJS/ PKP, InDesign, organizing various research training for a wide range of participants such as medical students, intern doctors, medical officers, residents, consultants, etc. As the inclusion of medical student, fellows, residents on editorial boards would create a pathway for continued activity in the editorial process after students complete their initial term.^2^ Existent resident editorial board fellowships have shown promising results, with Annals of Emergency Medicine fellowship participants remaining active in academic medicine.^3^ Being editorial support to JNMA, also aided in my academics and ignited my interest in research and publication.

As editorial support of JNMA, correcting the basic format of the articles and communication with the authors. Basic formatting included preparation of articles according to the JNMA guidelines, which included a lot of components. After being trained rigorously for one month and learning editorial work under the guidance of our seniors, we were introduced to the real editorial work of JNMA. Opponents to medical student inclusion to the editorial board may posit that students have not learned enough science to contribute meaningfully to the editorial process. But early exposure of students quickly improves each student's understanding of research and scientific writing. Thus this further supports that medical students will benefit from formal inclusion in the editorial process.^2^ JNMA was published bimonthly, after inclusion into the team, I realized there were so many works done behind the scenes for publication of a single issue of the journal. Being the youngest member of the editorial team of JNMA, learning from everything became my universal rule.

Various training programs were organized under the banner of the Journal of Nepal Medical Association with guidance from our Editor-In-Chief sir. Responsibility for the management of different components of a single training program was given among the editorial team members. For successful management of even a single day training program, it requires a lot of hard work. Different components of the program should come together for the smooth running of the program. Everyone in the team was assigned a different task of the training program. After executing the different assigned task for the training program, throughout the day, the post-program review was done. Various research training was organized inside as well as outside Kathmandu valley. Organising training programs outside the valley was a challenge itself. With all logistic support from the office of the Journal of Nepal Medical Association as well as the local organizing body of the Nepal Medical Association, we were able to organize and successfully complete the training programs. Feedbacks from the participants were discussed among the team members and revision of guidelines for program execution was done. While training has proven them to be a nice opportunity to learn and teach others, beyond Medical School and beyond Internship were the programs targeted to medical student and interns doctor. Our editorial team deliver the contents to the participants under the sub-heading of research and publication, future career and personality development.

Along with training and editorial work, information sessions were organized for different career pathways after graduation from medical school for members of the editorial team. Various career pathways were discussed and an experience sharing session was organized. Exploration of each career pathways and decision about choosing one has always been a challenge even for the brightest minds.

Mentoring session with our seniors helped us to learn from their experiences and apply that knowledge in various small as well as big decisions of our life. Our mentors introduced a lot of new topics regarding our personality development. Various topics were included in discussion with our mentors which helped us in self-growth and shaping our personalities. After an introduction to various topics, we understood the importance of the holistic approach to excel in life.

Being a volunteer in the election for the executive body of Nepal Medical Association, helped us to learn and witness such a historic moment of selection of new executive committee. Different opportunities such as attending the various meeting at national levels, press conferences, an information session and many more, helped us to know more about the current scenario of the medical system in Nepal and our responsibilities as future doctors.

## FULFILLING RESPONSIBILITIES OF MEDICAL STUDENT AND ASSISTANT EDITOR

While being a medical student of the third year, I joined the editorial team of JNMA so I was a full-time student and trying to balance between my studies and editorial work. Medicine is itself regarded as one of the toughest subjects, on top of that, investing my time in research and publication, became challenging. Maintaining my grades and making the best out of my clinical rotations were my topmost priority through medical school. During my final year of MBBS at my medical college, in September of 2019, I was promoted to assistant editor of JNMA. After passing my final year of MBBS, I have started my one-year compulsory internship on May 2, 2021, at my hospital. Because medical editors bear some of the responsibility for the reliability of published research and, in turn, for the care of patients, the health of the public, allocation of resources, and standards of medical ethics and professional behaviour, editors must be trustworthy.^4^ Being a medical editor is tough but being a medical student and medical editor, at the same time, has proven to be challenging.

Students on editorial boards are always busy with their academic responsibilities. The structured, inflexible curriculum offers little time for a medical student to devote to the work of the journal.^5^ Efficient utilization of the time which I had became a must. With the little time, efficient study, editorial work and other aspects of my life were squeezed in that time. Many are fairly passionate at first for what they do as part of a journal's editorial board, but after a point, the need for appreciable benefits overpowers the passion, and interest wanes.^5^ For me, the interest grew stronger and stronger with each article I edited and every time, I learned new things.

Sometimes, authors find this adhere of editors to certain principles of publishing the article irritating. JNMA is a very author-friendly journal and has always been keen to help the authors through the best means we could provide. Few people will make their entire careers in medical editing, but many will spend years at it. If you have a creative spark and a love of language if you derive pleasure from helping others improve their work if you think that you can help inform the discussions about the future of medicine, and if you have thick skin, an editor's job is worth contemplating.^4^
